# 

**Published:** 1882-03

**Authors:** 


### INTERNATIONAL HAHNEMANNIAN ASSOCIATION.

By request, we republish the complete list of officers, bureaus, etc., of the Association. The next meeting will be held at Indianapolis, June, 1882. President, C. Pearson, M. D., Washington; Vice-President, T. F. Pomeroy M. D., New York City; Secretary, Walter M. James, M. D., Philadelphia; Cor. Secretary, E. W. Berridge, M. D., London; Treasurer, Ad. Lippe, M. D., Philadelphia.

Board of Censors:—E. Bayard, M. D., New York; H. Hatch, M. D., Washington; J. R. Haynes, M.D., Indianapolis; De Forest Hunt, M.D., Grand Rapids; T. T. Oliver, M. D., Chicago; Ad. Lippe, M. D., *Chairman*, Philadelphia.

### BUREAUS.

Clinical Medicine:—O. P. Baer, M. D., Richmond, Ind.; E. A. Ballard, M. D., Chicago; E. W. Berridge, M. D., London; R. R. Gregg, M. D., Buffalo, N. Y.; Wm. Jefferson Guernsey, M. D., Philadelphia; L. B. Wells, M. D., Utica, N. Y.; Geo. F. Foote, *Chairman*, Stamford, Conn.

Surgical Therapeutics:—J. A. Biegler, M. D., Rochester, N. Y.; E. Carleton, M. D., N. Y.; Adolph Fellger, M. D., Philadelphia; J. F. Griffin, M. D., Williamsport, Pa.; John Hall, M. D., Toronto; J. R. Haynes, M. D., Indianapolis; H. I. Ostrom, M. D., N. Y.; J. B. Bell, M. D., *Chairman*, Boston.

Obstetrics, Gynecology, etc.:—J. B. Gregg Custis, M. D., Washington; W. H. Kern, M. D., M'Keesport, Pa.; Laura Morgan, M. D., San Francisco; E. B. Nash, M. D, Cortland, N. Y.; Edward Rushmore, M. D., Plainfield, N. J.; C. Carleton Smith, M. D., Philadelphia; Julius Schmitt, M. D., Rochester, N. Y.; Titus L. Brown, M. D., *Chairman*, Binghamton, N. Y.

Materia Medica:—H. C. Allen, M. D., Ann Arbor; E. Bayard, M. D., N. Y.; G. Pompili, M. D., Rome, Italy; C. F. Nichols, M. D., Boston; John C. Roberte, M. D., New Utrecht, N. Y.; W. P. Wesselhœft, M. D., Boston; P. P. Wells, M. D., Brooklyn; Adolph Lippe, M. D., *Chairman*, Philadelphia.

VOL. II. MARCH, 1882. No. 3.

### THE HOMŒOPATHIC PHYSICIAN,

A MONTHLY JOURNAL OF MEDICAL SCIENCE.

"*Magna est veritas, et prævalebit.*"

E. J. LEE, M. D.
EDITOR.

CONTENTS:

(See inside of Cover.)

NOTICE.

***All articles for publication, books for review, business communications, etc., must be sent to Editor, 2109 Chestnut Street, Philadelphia.***

PHILADELPHIA:

### CONTRIBUTORS:

Drs. H. C. Allen, T. F. Allen, Edward Bayard, J. B. Bell, E. W. Berridge, Geo. H. Clark, A. C. Cowperthwaite, Ad. Fellger, Wm. J. Guernsey, W. A. Hawley, John Hall, W. M. James, W. H. Jenny, Ad. Lippe, C. Lippe, Malcolm MacFarlan, Thos. Moore, C. F. Nichols, C. Pearson, T. F. Pomeroy, Leila A. RenDell, C. Carleton Smith, P. P. Wells, Wm. P. Wesselhoeft, David Wilson, (London), T. P. Wilson, and many others.

Authors of accepted papers are furnished with copies of the number in which their articles appear; application for such copies to be made when sending papers. Any irregularity in the receipt of this Journal should be promptly reported to Editor.

